# Antifungal Activity of Essential Oil of* Eucalyptus camaldulensis* Dehnh. against Selected* Fusarium* spp.

**DOI:** 10.1155/2017/8761610

**Published:** 2017-01-03

**Authors:** Martin Muthee Gakuubi, Angeline W. Maina, John M. Wagacha

**Affiliations:** ^1^School of Biological Sciences, University of Nairobi, P.O. Box 30197-00100, Nairobi, Kenya; ^2^Department of Biology, Faculty of Science, Mwenge Catholic University, P.O. Box 1226, Moshi, Tanzania

## Abstract

The objective of this study was to evaluate the antifungal activity of essential oil (EO) of* Eucalyptus camaldulensis* Dehnh. against five* Fusarium* spp. commonly associated with maize. The essential oil had been extracted by steam distillation in a modified Clevenger-type apparatus from leaves of* E. camaldulensis *and their chemical composition characterized by gas chromatography mass spectrometry. Poisoned food technique was used to determine the percentage inhibition of mycelial growth, minimum inhibitory concentration, and minimum fungicidal concentration of the EO on the test pathogens. Antifungal activity of different concentrations of the EO was evaluated using disc diffusion method. The most abundant compounds identified in the EO were 1,8-cineole (16.2%), *α*-pinene (15.6%), *α*-phellandrene (10.0%), and p-cymene (8.1%). The EO produced complete mycelial growth inhibition in all the test pathogens at a concentration of 7-8 *μ*L/mL after five days of incubation. The minimum inhibitory concentration and minimum fungicidal concentration of the EO on the test fungi were in the range of 7-8 *μ*L/mL and 8–10 *μ*L/mL, respectively. These findings confirm the fungicidal properties of* E. camaldulensis *essential oils and their potential use in the management of economically important* Fusarium* spp. and as possible alternatives to synthetic fungicides.

## 1. Introduction 

*Fusarium *spp. are phytopathogenic fungi of great economic importance whose effects on agricultural production are well documented [1]. These fungi are ubiquitous in soils [2] and colonize crops in temperate and semitropical regions [3] as well as the tropics [4].* Fusarium* spp. cause a wide range of plant diseases such as wilts and cankers in many horticultural, field, and ornamental plants; root rots, stalk rots, and ear and kernel rot in maize [5, 6]. Maize ear and kernel rot caused by* Fusarium* spp. is one of the most important diseases that affect maize production resulting in decrease in grain yield and quality due to contamination with various mycotoxins such as fumonisins, deoxynivalenol, nivalenol, and zearalenone [6, 7].

The most important* Fusarium *species that infect maize include* F. graminearum, F. oxysporum, F. sporotrichioides*,* F. verticillioides, *and* F. proliferatum *[2].* Fusarium* spp. produce an array of mycotoxins such as fumonisins, trichothecenes, zearalenone T-2 toxin, and HT-2 toxin that contaminate agricultural products resulting in huge economic losses [7, 8]. It is estimated that more than 50% of maize grain is lost as a result of infection by* Fusarium* spp. and subsequent mycotoxin contamination [9]. Furthermore, mycotoxins produced by these fungi pose serious health concerns for humans and livestock [10]. Exposure to* Fusarium* mycotoxins such as trichothecenes, zearalenone, and fumonisins is known to cause serious human and livestock illnesses such as anorexia, depression, gastroenteritis, immunological dysfunction, and haematoxicity while some of the toxins are potentially carcinogenic [11, 12].

Three main approaches, namely, physical, biological, and chemical treatments, have been used in the control of fungal growth, mycotoxin biosynthesis, and food contamination [13]. However, the modern system of crop protection against mycotoxigenic fungi has primarily relied on chemical methods especially the use of synthetic fungicides and chemical preservatives [14, 15]. Overreliance on synthetic fungicides in the control and management of mycotoxigenic fungi is not without serious environmental, ecological, and health concerns. Some of the drawbacks associated with the use of synthetic chemicals include development of resistance among the target microorganisms, toxicity to humans, animals and other nontarget organisms, and long environmental retention period leading to residual toxicities and environmental pollution [15, 16]. These among other factors have led to an increase in the quest for alternatives to synthetic fungicides for management of mycotoxigenic fungi.

For many years, plants and plant-derived metabolites have served as the starting point for the discovery and development of new antimicrobial agents. Phytochemicals have been recognized as some of the most promising compounds for the development of novel ecofriendly phytofungicides. Indeed, the need to develop plant-based fungicides as alternatives to synthetic chemicals has become a matter of priority among scientists globally [17]. A variety of plant extracts and secondary metabolites such as essential oil, tannins, alkaloids, and flavonoids have been reported to have promising in vitro fungitoxic activities against a range of fungi [18]. The primary advantages of using plant-derived antimicrobials in comparison to synthetic chemicals are their low mammalian toxicity, high degradability, multiple mechanisms of action, and fewer incidences of the numerous side effects often associated with synthetic chemicals [16]. Numerous research reports have highlighted the bioactive properties of plant essential oil against a wide range of economically important plant pathogens [19, 20].

*Eucalyptus* (Myrtaceae) represents an important genus of about 800 species, hybrids, and varieties that are native to Australia and Tasmania [21]. Members of this genus are known as important reservoir of a wide range of secondary metabolites many of which harbor a diverse range of biological activities [22, 23].* Eucalyptus camaldulensis* Dehnh. commonly known as the river red gum has its origin in the Australian mainland [24]. It is a highly adaptable tree with ability to tolerate extremes of drought and soil salinity. It is a medium-sized, fast-growing tree that can reach heights of 25–30 meters and one-meter diameter at breast height (D.B.H) but can also grow to heights of up to 50 meters [25].

Numerous pharmacological and phytochemical studies have reported antifungal potential of various extracts from* E. camaldulensis. *Aqueous and organic extracts of* E. camaldulensis* have been reported to have antifungal activity against* Fusarium solani* [26]. Methanolic extracts of* E. camaldulensis *have also been found to be active against* Alternaria alternata*, a phytopathogenic fungus that is responsible for causing leaf spot and other diseases on over 380 host species [27]. In addition to organic and aqueous extracts, essential oil of* E. camaldulensis *has been studied for their antifungal activity against a wide range of economically important phytopathogenic fungi including* Penicillium digitatum, Aspergillus flavus, Colletotrichum gloeosporioides, Pythium ultimum, Rhizoctonia solani, Bipolaris sorokiniana*,* F. graminearum, *and* F. sporotrichioides *[20, 28]. The objective of this study was to evaluate the antifungal activity of essential oil of* E. camaldulensis* against five economically important* Fusarium* spp. commonly associated with maize.

## 2. Materials and Methods

### 2.1. *Eucalyptus camaldulensis* Essential Oil

The essential oil of* Eucalyptus camaldulensis* Dehnh. was originally steam-distilled from leaves of* E. camaldulensis* collected from a plantation within Maseno area (0°0′10.39′′S, 34°36′71′′E; 1524 M.A.S.L) in Kisumu County, Kenya, from October to November, 2015 [29]. A subsample of the collected plant materials was prepared, packaged, and stored according to the herbarium rules and regulations. This sample was later taken to the herbarium at the School of Biological Sciences, University of Nairobi, Kenya, for identification, authentication, and further taxonomic studies. Authentication of the collected plant materials was performed by a plant taxonomist at the School of Biological Sciences, University of Nairobi, and a voucher specimen (MMG2015/03) deposited at the university's herbarium. The EO whose chemical composition was previously established using gas chromatography mass spectrometry contained majorly a mixture of monoterpenes and sesquiterpenes and their analogues, namely, oxygenated monoterpenes and sesquiterpenes. The most abundant constituents in the EO were 1,8-cineole (16.2%), *α*-pinene (15.6%), and *α*-phellandrene (10%) [29]. After extraction, the essential oil was stored at −20°C in Microbiology Laboratory at the School of Biological Sciences, University of Nairobi, until when it was required for the antimicrobial bioassays.

### 2.2. Description and Retrieval of Fungal Test Pathogens

Isolates of five plant pathogenic* Fusarium* species,* F. oxysporum, F. solani, F. verticillioides, F. proliferatum, *and* F. subglutinans*, were used as test pathogens. The five test pathogens were originally isolated from infected maize kernels and stored at −20°C at the Culture Collection Center, Mycology Laboratory, School of Biological Sciences, University of Nairobi, Kenya. To obtain pure single-spore cultures, hyphal tips from the fungal cultures were subcultured onto Potato Dextrose Agar (PDA) and incubated at 25°C in the dark for 14 days to induce sporulation. Confirmation of the identity of the resultant cultures was based on cultural and morphological characteristics [2, 30].

### 2.3. Determination of the Antifungal Activity of* Eucalyptus camaldulensis* Essential Oil

Assessment of the antifungal activity of the essential oil was carried out using the poisoned food technique as described by Adjou et al. [31]. Specific initial concentrations (1, 2, 3, 4, 5, 6 7, 8, 9, and 10 *μ*L/mL) were prepared by adding appropriate amount of* E. camaldulensis* essential oil containing 0.5% (v/v) of Tween 80 to cooled molten PDA (45°C) followed by manual rotation in a sterile Erlenmeyer flask to disperse the oil in the medium. Twenty milliliters of the medium was dispensed into sterile petri dishes (9 cm in diameter) with enough care taken to avoid trapping of air bubbles. The medium was allowed to solidify at room temperature (23 ± 2°C) for about one hour. Agar discs with mycelia (6 mm in diameter) were cut from the periphery of actively growing regions of the 7-day-old pure cultures using a sterile cork borer and aseptically inoculated at the center of the petri plates. Control plates (without the essential oil) were inoculated following the same procedure. Three replicates were maintained for each treatment and the plates were incubated at 28°C. The fungal colony diameter readings were taken after three and five days of incubation. The percentage inhibition of the mycelial growth of the test fungi by the essential oil was calculated using the formula by Philippe et al. [32].

(1) $$\begin{array}{c} \text{Inhibition of mycelial growth }\left(\;\%\;\right)=\frac{dc-dt}{dc}\times 100, \end{array}$$

where *dc* is mean diameter of colony in the control sample, 6 mm, and *dt* is mean diameter of colony in the treated sample, 6 mm.

### 2.4. Determination of Minimum Inhibitory Concentration and Minimum Fungicidal Concentration

Minimum inhibitory concentration (MIC) was defined as the lowest concentration of essential oil at which no growth occurred; that is, there was no change in the mycelia disc diameter. To establish whether the essential oil had biocidal effect on the test fungi, minimum fungicidal concentrations (MFCs) of oils on the test fungi were assessed as follows. The inhibited fungal discs of the oil treated plates were reinoculated into freshly prepared PDA petri plates and their growth revival observed after incubation for 72 hours at 28°C. Minimum fungicidal concentration was taken as the lowest concentration of the oil at which no growth occurred on the plates after subculturing [31].

### 2.5. Evaluation of Antifungal Activity of Essential Oil at Different Concentrations

Disc diffusion method was used to assess the antifungal activity of different concentrations of the EO against the test fungi as described by Clara et al. [19]. Two hundred microliters of spore suspension (approximately 10^8^ spores/mL) was uniformly spread using a sterile L-shaped glass rod on 9 cm diameter Petri plates containing PDA medium. Serial dilution was used to prepare seven concentrations (i.e. 100, 50, 25, 12.5, 6.25, 3.13, and 1.56% v/v) of essential oil in dimethyl sulfoxide (DMSO). Sterile Whatman filter paper discs (no. 1, 6 mm in diameter) were impregnated with 10 *μ*L of different essential oil concentrations and aseptically placed at the center of the inoculated culture plates using a sterile pair of forceps. The plates were placed in a refrigerator at 4°C for 2 hours to allow the essential oil to diffuse into the agar and then incubated at 28°C for five days. At the end of the incubation period, antifungal activity was evaluated by measuring the zone of inhibition (mm) against the test fungi. The fungicide Apron Star® and DMSO were used as positive and negative controls, respectively. The tests were conducted in triplicate.

### 2.6. Data Analysis

The data were analyzed using the PROC ANOVA procedure of GENSTAT version 15 and significant differences among the means compared using Fisher's protected LSD at 5% probability level. Linear regression analysis was performed to establish any correlations among different concentrations of the essential oil and their overall antifungal activity.

## 3. Results

### 3.1. Antifungal Activity of* Eucalyptus camaldulensis* Essential Oil

**Figure 1:**
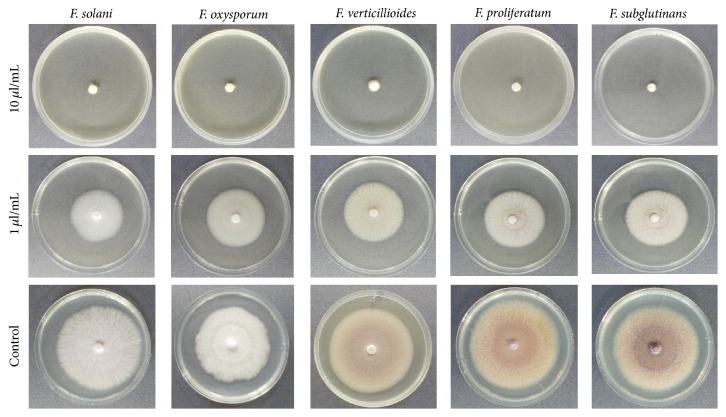
Inhibition of mycelia growth in* F. solani*,* F. oxysporum, F. verticillioides, F. proliferatum,* and* F. subglutinans *by essential oil of* E. camaldulensis* at 1 and 10 *μ*L/mL after five days of incubation.

Essential oil of* E. camaldulensis *inhibited mycelial growth in the five test fungi,* F. oxysporum*,* F. solani*,* F. verticillioides*,* F. proliferatum, *and* F. subglutinans.* In all the test fungi, complete mycelial growth inhibition was observed at an essential oil concentration of 10 *μ*L/mL (Figure 1).

*Eucalyptus camaldulensis *essential oil had a significantly (*p* ≤ 0.05) higher inhibitory effect on* F. solani *and* F. Proliferatum *than on* F. oxysporum*,* F. verticillioides,* and* F. subglutinans *at a concentration of 1 *μ*L/mL after three days of incubation (Table 1). The range of mycelial growth inhibition was between 44.4% and 100%. At a concentration of 6 *μ*L/mL, the EO completely inhibited the mycelial growth of* F. solani*,* F. oxysporum,* and* F. proliferatum.* However, complete inhibition of* F. subglutinans* and* F. verticillioides *was observed at a concentration of 8 *μ*L/mL (Table 1).

### 3.2. Minimum Inhibitory Concentration and Minimum Fungicidal Concentration

Inhibition of the mycelial growth of the test fungi by* E. camaldulensis *EO after five days of incubation ranged from 31.5% to 100% (Table 2). The highest and lowest rates of mycelial growth inhibition by the EO at a concentration of 1 *μ*L/mL were observed in* F. proliferatum* (46%) and* F. oxysporum* (31.5%), respectively. The inhibition of mycelial growth in the five test fungi at an EO concentration of 1 *μ*L/mL was significantly (*p* ≤ 0.05) different. By the 7th day of incubation, mycelia of the test fungi with the exception of* F*.* oxysporum *had overgrown the diameter of the petri plates. Therefore, evaluation of growth inhibition percentage (GI%) was not possible after this period.

The minimum inhibitory concentrations (MICs) of the EO of* E. camaldulensis *on the test pathogens were in the range of 7-8 *μ*L/mL while the minimum fungicidal concentrations (MFCs) were in the range of 8–10 *μ*L/mL (Table 2). The lowest MIC value (7 *μ*L/mL) was observed in* F. oxysporum *while the other* Fusarium* isolates had MICs value of 8 *μ*L/mL. The essential oil of* E. camaldulensis *showed fungicidal effect on four out of the five studied fungi, namely,* F. oxysporum*,* F. verticillioides*,* F. proliferatum, *and* F. subglutinans*. However, the EO did not show any fungicidal activity against* F. solani *at any of the essential oil concentrations tested in the study.

### 3.3. Activity of Different Concentrations of* Eucalyptus camaldulensis* Essential Oil on* Fusarium* spp. 

The essential oil of* E. camaldulensis* exhibited a concentration-dependent activity against the test fungi (Table 3). Overall, as the concentration of the essential oil increased, the activity against the test fungi increased represented by an increase in the diameter of the inhibition zones. However, there were some instances where more dilute essential oil produced larger inhibition zones than the less dilute oil. The highest activity of undiluted crude EO was observed in* F. solani *with a mean inhibition zone of 20.33 mm. The mean inhibition zones of essential oil at concentrations of 50, 25, and 12.5% were 22.3, 17.4 and 7.3 mm, respectively, in* F. solani*. The lowest activity of the EO at a concentration of 100% occurred in* F. proliferatum* where a mean inhibition zone of 12.00 mm was recorded. The highest concentrations of* E. camaldulensis* EO at which no appreciable inhibition zones were observed (inhibition zone of ≤ 6 mm) were 6.25% for* F. solani *and* F. verticillioides*, 3.13% for* F. proliferatum* and* F. subglutinans, *and 1.56% for* F. oxysporum*.

### 3.4. Dose-Response Effect of the Essential Oil on the Growth of* Fusarium* spp. 

**Figure 2:**
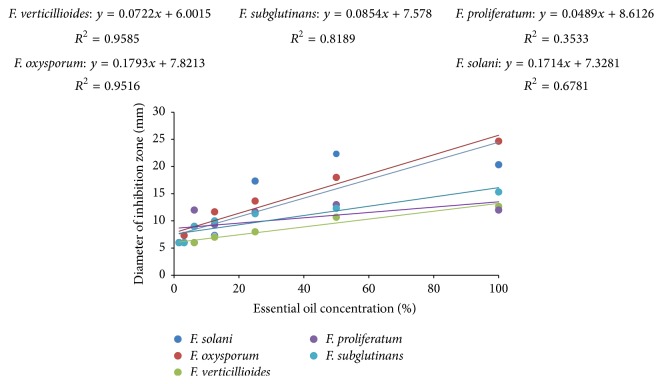
A dose-response curve of inhibition zone diameter (mm) against the concentration percentage of* E. camaldulensis* essential oil.

The results of regression analysis showed that generally essential oil of* E. camaldulensis* inhibited growth of the test* Fusarium* spp. in a dose-dependent manner. Thus, as the essential oil concentration increased, the antifungal activity against the test fungi increased (Figure 2). There was a significant correlation (*p* ≤ 0.05) between the tested essential oil concentrations and mean inhibition zones in* F. oxysporum *(*R*^2^ = 0.96; *p* < 0.001),* F. solani *(*R*^2^ = 0.68; *p* = 0.023),* F. subglutinans *(*R*^2^ = 0.82; *p* = 0.005), and* F. verticillioides *(*R*^2^ = 0.96; *p* < 0.001). An exception to this pattern was however observed in* F. proliferatum* (*R*^2^ = 0.35; *p* = 0.159) where no significant correlation (*p* ≥ 0.05) was observed between the concentration of the EO and the mean inhibition zones.

## 4. Discussion

*Eucalyptus camaldulensis *essential oil had activity against the five test* Fusarium* species, namely,* F. oxysporum*,* F. solani*,* F. verticillioides*,* F. proliferatum, *and* F. subglutinans*. However, the antifungal activity of crude essential oil varied among the test pathogens. The findings of the current study concur with reports from previous studies on different levels of antifungal activity of essential oil of* E. camaldulensis* of varied chemical profiles against a diverse group of plant pathogenic fungi. A study was carried out to evaluate mycelial growth suppression action of* E. camaldulensis *EO against postharvest pathogenic fungi;* Penicillium digitatum, Aspergillus flavus, Colletotrichum gloeosporioides, *and soil borne pathogenic fungi;* Pythium ultimum, Rhizoctonia solani, *and* Bipolaris sorokiniana* [20]. There was complete inhibition of mycelial growth in* P. ultimum *and* R. solani *by the four tested EO concentrations (i.e., 25, 50, 75, and 100%) after 30 days of incubation. The EO had complete inhibition of* B. sorokiniana *and* C. gloeosporioides *only until 5 days while no mycelial growth inhibition was recorded in* P. digitatum *and* A. flavus.*

In a study to investigate the antifungal activity of* E. camaldulensis *EO against* F. graminearum *and* F. sporotrichioides*, the antifungal index increased with increase in concentration of the essential oil and varied between 0% and 34.1% in* F. sporotrichioides *and between 29.1% and 41.8% in* F. graminearum *[28]. In another study, the inhibitory activity of* E. camaldulensis *EO against a wide range of household molds, wood rot fungi, and plant pathogenic fungi such as* Chaetomium globosum*,* F. oxysporum*,* Aspergillus niger*,* Thanatephorus cucumeris,* and* Rhizopus oryzae* was investigated [33]. The essential oil induced 84 and 100% inhibition of the mycelial growth of* F. oxysporum* and* T. cucumeris* at a concentration of 5 mg/mL and 100% inhibition of* C. globosum* at a concentration of 10 mg/mL. Essential oil of* E. camaldulensis *has also been reported to have activity against three soil-borne fungi, namely,* Glomerella graminicola*,* Phoma sorghina,* and* F. moniliforme* [34].

In the current study, the minimum fungicidal concentration values were obtained for four out of the five studied fungi, namely,* F. oxysporum*,* F. verticillioides*,* F. proliferatum, *and* F. subglutinans*. The EO in all the ten studied concentrations did not produce fungicidal activity in* F. solani.* To obtain MFC of the oils against* F. solani* would therefore require an analysis of much higher EO concentrations. Both the poisoned food and disc diffusion bioassays revealed that the EO of* E. camaldulensis *inhibited the growth of the test fungi in a dose-dependent manner. There are many reports in literature of concentration-dependent antifungal activity of essential oil whereby the colony diameters increase with decrease in the concentration of EO (poisoned food bioassay) or the diameters of the inhibition zone increase with increase in the concentration of the essential oil (disk diffusion assay) [35, 36]. Some exceptional instances were however observed in the current study such as in the case of* F. solani *and* F. proliferatum* where undiluted EO produced smaller inhibition zones in comparison to diluted essential oil. This could explain the lack of a linear correlation between the essential oil concentrations and growth inhibition of* F. proliferatum* that was observed in the disc diffusion bioassay. Instances where more concentrated EO produce smaller inhibition zones in comparison to less concentrated oil have been reported in literature and are attributed to the fact that dilute EO diffused more easily in the agar medium (i.e., aqueous environment) than the undiluted or less dilute EO [37, 38]. Furthermore, higher rate of polymerization of the undiluted essential oil may lead to reduced antimicrobial activity and hence smaller inhibition zones [38].

## 5. Conclusion

Growth inhibitory potential of plant extracts and secondary metabolites such as essential oil against microorganisms of economic importance remain a focal priority area for future research. The essential oil of* E. camaldulensis *completely inhibited mycelial growth of the five isolates of* Fusarium* spp. at a concentration range between 7 and 8 *μ*L/mL after five days of incubation. The study therefore confirms the fungicidal nature of* E. camaldulensis *essential oil and the potential uses of this oil as an alternative to chemical fungicides or as template for synthesis of new and more effective fungicides for management of plant pathogenic* Fusarium* species. However, further studies should be conducted to evaluate the efficacy of* E. camaldulensis* essential oil against phytopathogenic fungi under field conditions.

## Supplementary Material

Gas chromatography mass spectrometry (GC/MS) analysis of the essential oil of Eucalyptus camaldulensis Dehnh. identified a total of fifty-four compounds corresponding to 95% of the essential oil. The essential oil contained majorly a mixture of monoterpenes and sesquiterpenes hydrocarbons. The most abundant monoterpene was 1,8-Cineole (16.2%) followed closely by α-pinene (15.6%) while the least was δ-2- carene 0.2%. Sesquiterpenes concentration on the other hand ranged from 0.2 - 2.1% with iso-leptospermone and (E)-caryophyllene being the most abundant within this group of compounds accounting for 2.2% and 1.6%, respectively.

## Figures and Tables

**Table 1:** Mycelial growth inhibition rate (%) of essential oil of *E. camaldulensis* on five *Fusarium* spp. after three days of incubation.

Essential oil concentration (*μ*L/mL)	Mycelial growth inhibition (%)				
	*F. solani*	*F. oxysporum*	*F. verticillioides*	*F. proliferatum*	*F. subglutinans*
(1)	63.44 ± 1.08^j^	44.44 ± 2.14^l^	45.56 ± 2.94^l^	56.57 ± 1.01^k^	46.68 ± 1.45^l^
(2)	77.42 ± 1.63^ef^	72.84 ± 3.27^g–i^	53.33 ± 1.93^k^	64.65 ± 2.02^j^	62.07 ± 1.99^j^
(3)	87.10 ± 1.86^c^	86.42 ± 2.46^c^	68.89 ± 2.94^i^	75.76 ± 1.75^f–h^	72.41 ± 1.99^hi^
(4)	93.55 ± 0.86^b^	91.36 ± 1.24^b^	76.76 ± 1.93^e–g^	86.87 ± 1.01^c^	81.61 ± 1.14^d^
(5)	94.62 ± 1.08^b^	95.06 ± 1.24^b^	80.00 ± 1.93^de^	94.95 ± 1.01^b^	91.95 ± 1.15^b^
(6)	100^a^	100^a^	92.22 ± 1.11^b^	100^a^	93.10 ± 2.43^b^
(7)	100^a^	100^a^	94.44 ± 2.22^b^	100^a^	94.25 ± 2.30^b^
(8)	100^a^	100^a^	100^a^	100^a^	100^a^
(9)	100^a^	100^a^	100^a^	100^a^	100^a^
(10)	100^a^	100^a^	100^a^	100^a^	100^a^

Values are mean ± standard error of the mean for bioassay conducted in triplicate. Means followed by the same letter(s) are not significantly different (multivariate analysis, Fisher's protected LSD at *p* ≤ 0.05).

**Table 2:** Growth inhibition rates (%) with MIC and MFC concentrations of *E. camaldulensis *essential oil against five *Fusarium* species after five days of incubation.

Essential oil concentration (*µ*L/mL)	Mycelial growth inhibition (%)				
	*F. solani*	*F. oxysporum*	*F. verticillioides*	*F. proliferatum*	*F. subglutinans*
(1)	35.22 ± 1.66^t^	31.48 ± 2.13^u^	40.32 ± 1.61^s^	46.03 ± 1.83^r^	32.20 ± 1.96^tu^
(2)	53.46 ± 1.26^q^	54.94 ± 1.63^pq^	57.53 ± 2.34^op^	60.85 ± 1.06^no^	51.98 ± 2.04^q^
(3)	44.65 ± 2.27^r^	61.73 ± 163^mn^	62.90 ± 0.93^mn^	65.08 ± 1.59^lm^	57.63 ± 1.96^op^
(4)	75.47 ± 2.18^hi^	70.99 ± 163^jk^	67.20 ± 2.43^l^	74.07 ± 2.31^ij^	68.36 ± 2.04^kl^
(5)	78.62 ± 1.66^gh^	80.25 ± 1.63^g^	76.88 ± 1.42^g–i^	79.89 ± 1.4^g^	86.44 ± 0.98^d–f^
(6)	84.28 ± 1.66^f^	89.51 ± 1.63^d^	84.95 ± 1.04^f^	85.71 ± 0.92^ef^	88.70 ± 1.13^de^
(7)	93.71 ± 1.66^b^	100^a○^	87.63 ± 1.42^d–f^	93.12 ± 1.40^bc^	89.83 ± 0.98^cd^
(8)	100^a○^	100^a^	100^a○^	100^a○^	100^a○*∗*^
(9)	100^a^	100^a*∗*^	100^a*∗*^	100^a^	100^a^
(10)	100^a^	100^a^	100^a^	100^a*∗*^	100^a^

Values are mean ± standard error of the mean for bioassay conducted in triplicate. Means followed by the same letter(s) are not significantly different (multivariate analysis, Fisher's protected LSD at *p* ≤ 0.05).

^○^Minimum inhibitory concentration; ^*∗*^minimum fungicidal concentration.

**Table 3:** Inhibition zone (mm) on test fungi by different concentrations of *Eucalyptus camaldulensis* essential oil after five days of incubation.

Fungi	Essential oil concentration (% v/v)							Apron star (+ control)
	100	50	25	12.5	6.25	3.13	1.56	
*F. solani*	20.33 ± 1.20^n^	22.33 ± 1.20^n^	17.43 ± 1.20^lm^	7.31 ± 0.33^a–c^	6.00^a^	6.00^a^	6.00^a^	15.00
*F. oxysporum*	24.67 ± 1.20^o^	18.00 ± 1.53^m^	13.67 ± 0.33^jk^	11.67 ± 0.33^g–j^	9.00 ± 0.58^b–e^	7.33 ± 0.33^a–c^	6.00^a^	25.33
*F. verticillioides *	12.67 ± 1.20^h–j^	10.67 ± 0.67^e–h^	8.00 ± 1.84^a–d^	7.00 ± 0.57^ab^	6.00^a^	6.00^a^	6.00^a^	31.67
*F. proliferatum*	12.00 ± 0.58^g–j^	13.00 ± 1.53^ij^	11.67 ± 0.88^g–j^	9.33 ± 0.33^c–f^	12.00 ± 0.58^g–j^	6.00^a^	6.00^a^	34.33
*F. subglutinans*	15.33 ± 0.88^kl^	12.33 ± 1.20^h–j^	11.33 ± 0.33^f–i^	10.00 ± 0.58^d–g^	9.00 ± 0.58^b–e^	6.00^a^	6.00^a^	36.33

Values are mean ± standard error of the mean for bioassay conducted in triplicates. Means followed by the same letter(s) are not significantly different (multivariate analysis, Fisher's protected LSD at *p* ≤ 0.05).

## References

[1] Maina P. K., Okoth S., Monda E. O. (2009). Impact of land use on distribution and diversity of *Fusarium* species in Taita, Kenya. *Tropical and Subtropical Agroecosystems*.

[2] Leslie J. F., Summerell B. A. (2006). *The Fusarium Laboratory Manual*.

[3] Logrieco A., Mulè G., Moretti A., Bottalico A. (2002). Toxigenic *Fusarium* species and mycotoxins associated with maize ear rot in Europe. *European Journal of Plant Pathology*.

[4] Manshor N., Rosli H., Ismail N. A., Salleh B., Zakaria L. (2012). Diversity of *Fusarium* species from highland areas in Malaysia. *Tropical Life Sciences Research*.

[5] Ma L.-J., Geiser D. M., Proctor R. H. (2013). *Fusarium* pathogenomics. *Annual Review of Microbiology*.

[6] Duan C., Qin Z., Yang Z. (2016). Identification of pathogenic *Fusarium*spp. causing maize ear rot and potential mycotoxin production in China. *Toxins*.

[7] Glenn A. E. (2007). Mycotoxigenic *Fusarium* species in animal feed. *Animal Feed Science and Technology*.

[8] Streit E., Schatzmayr G., Tassis P. (2012). Current situation of mycotoxin contamination and co-occurrence in animal feed—focus on Europe. *Toxins*.

[9] Uzma S., Shahida A. (2007). The screening of seven medicinal plants for artificial activity against seed borne fungi of maize seeds. *Pakistan Journal of Botany*.

[10] Bankole S., Schollenberger M., Drochner W. (2006). Mycotoxins in food systems in Sub Saharan Africa: a review. *Mycotoxin Research*.

[11] Yazar S., Omurtag G. Z. (2008). Fumonisins, trichothecenes and zearalenone in cereals. *International Journal of Molecular Sciences*.

[12] Wan L. Y. M., Turner P. C., El-Nezami H. (2013). Individual and combined cytotoxic effects of *Fusarium toxins* (deoxynivalenol, nivalenol, zearalenone and fumonisins B1) on swine jejunal epithelial cells. *Food and Chemical Toxicology*.

[13] Fernanda C., Chalfoun S. M., Siqueira V. M., Botelho D. M., Lima N., Batista L. R. (2012). Evaluation of antifungal activity of essential oils against potentially mycotoxigenic *Aspergillus flavus* and *Aspergillus parasiticus*. *Revista Brasileira de Farmacognosia*.

[14] Avasthi S., Gautam A. K., Bhadauria R. (2010). Antifungal activity of plant products against *Aspergillus niger*: a potential application in the control of a spoilage fungus. *Biological Forum*.

[15] Paster N., Barkai-Golan R. (2008). Mouldy fruits and vegetables as a source of mycotoxins: part 2. *World Mycotoxin Journal*.

[16] Raja N. (2014). Botanicals: sources for eco-friendly biopesticides. *Journal of Biofertilizers and Biopesticides*.

[17] Reddy C. S., Reddy K. R. N., Prameela M., Mangala U. N., Muralidharan K. (2007). Identification of antifungal component in clove that inhibits *Aspergillus* spp. colonizing rice grains. *Journal of Mycology and Plant Pathology*.

[18] Anjorin T. S., Salako E. A., Makun H. A., Makun H. A. (2013). Control of toxigenic fungi and mycotoxins with phytochemicals: potentials and challenges. *Mycotoxin and Food Safety in Developing Countries*.

[19] Clara C., Matasyoh J. C., Wagara I. N., Nakuvuma J. (2013). Antifungal activity of *Monathotaxis littoralis* essential oil against mycotoxigenic fungi isolated from maize. *International Journal of Microbiology Research and Reviews*.

[20] Katooli N., Maghsodlo R., Razavi S. E. (2011). Evaluation of eucalyptus essential oil against some plant pathogenic fungi. *Journal of Plant Breeding and Crop Science*.

[21] Mubarak E. E., Ali L. Z., Ahmed I. F., Ali A. B. (2015). Essential oil compositions and cytotoxicity from various organs of *Eucalyptus camaldulensis*. *International Journal of Agriculture and Biology*.

[22] Cheng S.-S., Huang C.-G., Chen Y.-J., Yu J.-J., Chen W.-J., Chang S.-T. (2009). Chemical compositions and larvicidal activities of leaf essential oils from two eucalyptus species. *Bioresource Technology*.

[23] Huang H.-C., Ho Y.-C., Lim J.-M., Chang T.-Y., Ho C.-L., Chang T.-M. (2015). Investigation of the anti-melanogenic and antioxidant characteristics of *Eucalyptus camaldulensis* flower essential oil and determination of its chemical composition. *International Journal of Molecular Sciences*.

[24] Sabas E., Nshubemuki L. (1988). *Eucalyptus camaldulensis* provenances for afforestation in Mwanza and Shinyanga regions of Tanzania. *Forest Ecology and Management*.

[25] Agroforestree Database Eucalyptus camaldulensis. http://www.worldagroforestry.org/treedb/AFTPDFS/Eucalyptus_camaldulensis.PDF.

[26] Bashir U., Tahira J. J. (2012). Evaluation of *Eucalyptus camaldulensis* against *Fusarium solani*. *International Journal of Agriculture and Biology*.

[27] Singh G., Gupta S., Sharma N. (2014). *In vitro* screening of selected plant extracts against *Alternaria alternate*. *Journal of Experimental Biology*.

[28] Mehani M., Salhi N., Valeria T., Ladjel S. (2014). Antifungal effects of essential oil of *Eucalyptus camaldulensi*s plant on *Fusarium graminearum* and *Fusarium sporotrichioides*. *International Journal of Current Research*.

[29] Gakuubi M. M. (2016). Steam distillation extraction and chemical composition of essential oils of *Toddalia asiatica* L. and *Eucalyptus camaldulensis* Dehnh. *Journal of Pharmacognosy and Phytochemistry*.

[30] Nelson P. E., Toussoun T. A., Marasas W. F. O. (1983). *Fusarium Species: An Illustrated Manual for Identification*.

[31] Adjou E. S., Kouton S., Dahouenon-Ahoussi E., Sohounhloue C. K., Soumanou M. M. (2012). Antifungal activity of *Ocimum canum* essential oil against toxinogenic fungi isolated from peanut seeds in post-harvest in Benin. *International Research Journal of Biological Sciences*.

[32] Philippe S., Souaïbou F., Guy A. (2012). Chemical Composition and Antifungal activity of essential oil of fresh leaves of *Ocimum gratissimum* from Benin against six mycotoxigenic fungi isolated from traditional cheese *wagashi*. *Research Journal of Biological Sciences*.

[33] Siramon P., Ohtani Y., Ichiura H. (2013). Chemical composition and antifungal property of *Eucalyptus camaldulensis* leaf oils from Thailand. *Records of Natural Products*.

[34] Somda I., Leth V., Sérémé P. (2007). Antifungal effect of *Cymbopogon citratus, Eucalyptus camaldulensis* and *Azadirachta indica* oil extracts on sorghum seed-borne fungi. *Asian Journal of Plant Sciences*.

[35] Rana I. S., Rana A. S., Rajak R. C. (2011). Evaluation of antifungal activity in essential oil of the *Syzygium aromaticum* (L.) by extraction, purification and analysis of its main component eugenol. *Brazilian Journal of Microbiology*.

[36] Aguiar R. W. D. S., Ootani M. A., Ascencio S. D., Ferreira T. P. S., Santos M. M. D., Santos G. R. D. (2014). Fumigant antifungal activity of *Corymbia citriodora* and *Cymbopogon nardus* essential oils and citronellal against three fungal species. *The Scientific World Journal*.

[37] Hood J. R., Wilkinson J. M., Cavanagh H. M. A. (2003). Evaluation of common antibacterial screening methods utilized in essential oil research. *Journal of Essential Oil Research*.

[38] Osée Muyima N. Y., Nziweni S., Mabinya L. V. (2004). Antimicrobial and antioxidative activities of *Tagetes minuta*, *Lippia javanica* and *Foeniculum vulgare* essential oils from the Eastern Cape Province of South Africa. *Journal of Essential Oil Bearing Plants*.

