# Supplementary material for: Antifungal Activity of Essential Oil of Eucalyptus camaldulensis Dehnh. against Selected Fusarium spp
Source: Int J Microbiol. 2017 Jan 3;2017:8761610. doi: 10.1155/2017/8761610 (PMC5239988; doi:10.1155/2017/8761610)
Supplement: Supplementary file 1 — Gas chromatography mass spectrometry (GC/MS) analysis of the essential oil of Eucalyptus camaldulensis Dehnh. identified a total of fifty-four compounds corresponding to 95% of the essential oil. The essential oil contained majorly a mixture of monoterpenes and sesquiterpenes hydrocarbons. The most abundant monoterpene was 1,8-Cineole (16.2%) followed closely by α-pinene (15.6%) while the least was δ-2- carene 0.2%. Sesquiterpenes concentration on the other hand ranged from 0.2 - 2.1% with iso-leptospermone and (E)-caryophyllene being the most abundant within this group of compounds accounting for 2.2% and 1.6%, respectively. [file 8761610.f1.pdf]

Compounds identified from the essential oil of *Eucalyptus camaldulensis* Dehnh.

No[b]	RT (min)	Compound Name	RI[b]	Concentration mean (%) ±SE
1	4.427	Isopentyl formate	714	1.1±0.01
2	6.211	2,4-dimethyl-3-Pentanone	779	0.3±0.02
3	9.411	2-methylpropyl- 2-methylpropanoate	903	0.9±0.01
4	9.838	α-Pinene	922	15.6±0.18
5	10.054	Camphene	932	1.8±0.01
6	10.645	β-Pinene	958	4.6±0.04
7	10.961	Myrcene	972	0.6±0.02
8	11.224	α-Phellandrene	984	10.0±0.11
9	11.476	δ-2-Carene	996	0.2±0.02
10	11.669	p-Cymene	1006	8.1±0.08
11	11.809	1,8-Cineole	1014	16.2±0.19
12	12.037	(E)-β- Ocimene	1028	0.2±0.02
13	12.143	dihydro-Tagetone	1034	0.2±0.02
14	12.277	ɣ-Terpinene	1042	4.4±0.03
15	12.500	2,6-dimethyl-3,5-Heptanedione	1056	0.4±0.02
16	12.739	Terpinolene	1070	1.9±0.01
17	12.777	α-Terpinene	1317	0.5±0.02
18	12.915	3,7-dimethyl-1,6-Octadien-3-ol	1081	0.5±0.02
19	12.997	3-methyl-Butanoic acid-3-methylbutyl ester	1086	0.6±0.02
20	13.044	n-Amyl isovalerate	1089	0.4±0.02
21	13.190	endo-Fenchol	1098	1.6±0.01
22	13.377	2,6-dimethyl-2,4,6-Octatriene	1109	1.4±0.01
23	13.634	(E) - Pinocarviol	1124	2.3±0.01
24	13.699	Camphor	1128	0.3±0.02
25	13.757	Camphene hydrate	1131	0.3±0.02
26	13.980	Pinocarvone	1144	0.8±0.01
27	14.079	Borneol	1150	2.9±0.01
28	14.237	Terpinen-4-ol	1159	2.0±0.01
29	14.483	α-Terpineol	1174	4.4±0.03
30	14.541	Myrtenol	1177	0.9±0.01
31	14.834	(E)-Carveol	1194	0.5±0.02
32	15.155	Cumin aldehyde	1215	0.3±0.02
33	15.202	Carvone	1218	0.2±0.02
34	15.267	Carvotanacetone	1222	0.6±0.02
35	15.840	(E)- Isosafrole	1261	0.3±0.02
36	15.980	Carvacrol	1270	0.7±0.02
37	16.536	1,5,5-Trimethyl-6-methylene-cyclohexene	1308	0.5±0.02
38	17.033	Isoledene	1343	0.2±0.02
39	17.080	α-Copaene	1347	0.3±0.02
40	17.361	Methyl eugenol	1367	0.3±0.02

41	17.548	α-Gurjunene	1380	0.6±0.02
42	17.700	(E)-Caryophyllene	1391	1.6±0.01
43	17.788	β-Gurjunene	1398	0.2±0.02
44	17.952	β-Selinene	1410	1.0±0.01
45	18.233	α-Guaiene	1432	0.7±0.01
46	18.350	Zonarene	1441	0.3±0.02
47	18.572	Δ-Selinene	1458	0.5±0.02
48	18.654	Viridiflorene	1464	1.1±0.01
49	18.859	ɣ- Muurolene	1480	0.2±0.02
50	18.952	δ-Cadinene	1487	0.5±0.02
51	19.555	ɣ-Eudesmol	1536	0.3±0.03
52	19.672	Spathulenol	1546	0.7±0.02
53	19.754	Globulol	1553	0.7±0.01
54	20.093	iso-Leptospermone	1581	2.2±0.01

[b]No = Peak numbers
[b]RI = Retention index
SE = Standard error of the mean
